# May-Thurner Syndrome: A Case Report and a Concise Review

**DOI:** 10.7759/cureus.16256

**Published:** 2021-07-08

**Authors:** Ahmed Al Sinani, Waleed Al Saadi, Salma Al Harthi, Mahmood Al Hajriy

**Affiliations:** 1 Internal Medicine, The Royal Hospital, Muscat, OMN; 2 Internal Medicine, Oman Medical Specialty Board, Muscat, OMN; 3 Interventional Radiology, The Royal Hospital, Muscat, OMN

**Keywords:** thromboembolism, cocket’s syndrome, iliac vein compression syndrome, deep vein thrombosis (dvt), pulmonary embolism (pe), may-thurner's syndrome, balloon angioplasty with stent

## Abstract

We report a case of a 31-year-oldman who presented to the hospital with extensive deep vein thrombosis (DVT) complicated by pulmonary embolism (PE) after a recent trauma and prolonged immobilization. He underwent contrast venography that revealed features of May-Thurner syndrome (MTS). He was managed with therapeutic anticoagulation, inferior vena cava filter placement, mechanical clot aspiration, catheter-directed thrombolytic therapy, and left common iliac vein stenting.

MTS is a vascular condition caused by the compression of the left common iliac vein by an overlying right common iliac artery against a vertebral body. This results in indolent endothelial changes secondary to the pulsating nearby artery as well as the compression increasing the susceptibility to venous thrombosis. Females are thought to be more prone to the condition due to the nature of their pelvic anatomy. Most patients are asymptomatic or present with unspecific symptoms, rendering the condition underdiagnosed. The gold standard diagnostic modality is contrast venography that reveals collaterals and a pressure gradient greater than 2 mmHg at rest across the stenotic region. Treatment is revolved around the removal of the thrombus along with the correction of the anatomical defect through interventional or surgical treatment to prevent a recurrence.

Untreated MTS complicated with DVT carries a risk of potentially life-threatening complications, such as PE, iliac vein rupture, retroperitoneal hematoma, or refractory DVT that is difficult to treat. Due to the chronicity of this syndrome, its management plan differs from that of other causes of DVT. Proper identification of MTS carries a positive outcome in treating DVT secondary to MTS. Here we are going to discuss a case diagnosed with MTS complicated by saddle PE outlying the possible pathophysiology, clinical manifestation, diagnostic tools, and management of complicated MTS.

## Introduction

Deep vein thrombosis (DVT) is a common complication in many individuals who have the propensity for thrombosis. Virchow triad describes three major elements that favor venous thrombosis, namely blood stasis, hypercoagulable state, and endothelial injury. Usually, patients who develop venous thrombosis have identifiable risk factors that can cause an underlying pathology to be overlooked, increasing the chances of thrombosis and its recurrence [1]. This case, for example, was bed-bound for two weeks after sustaining a trauma. The complexity and extensivity of the case’s DVT formation made us think of the possible underlying pathology. In May-Thurner syndrome (MTS), the right common iliac artery is overlaying the left common iliac vein against the vertebral body. The pulsating right common iliac artery results in the slow formation of endothelial damage in the vein, contributing to thrombosis formation. MTS is an underrecognized condition that has a different management scheme from that of other common causes of venous thrombosis to prevent a recurrence. Hence, it is of great significance to properly identify the condition for optimal outcomes to reduce morbidity and mortality [1].

## Case presentation

**Figure 1:**
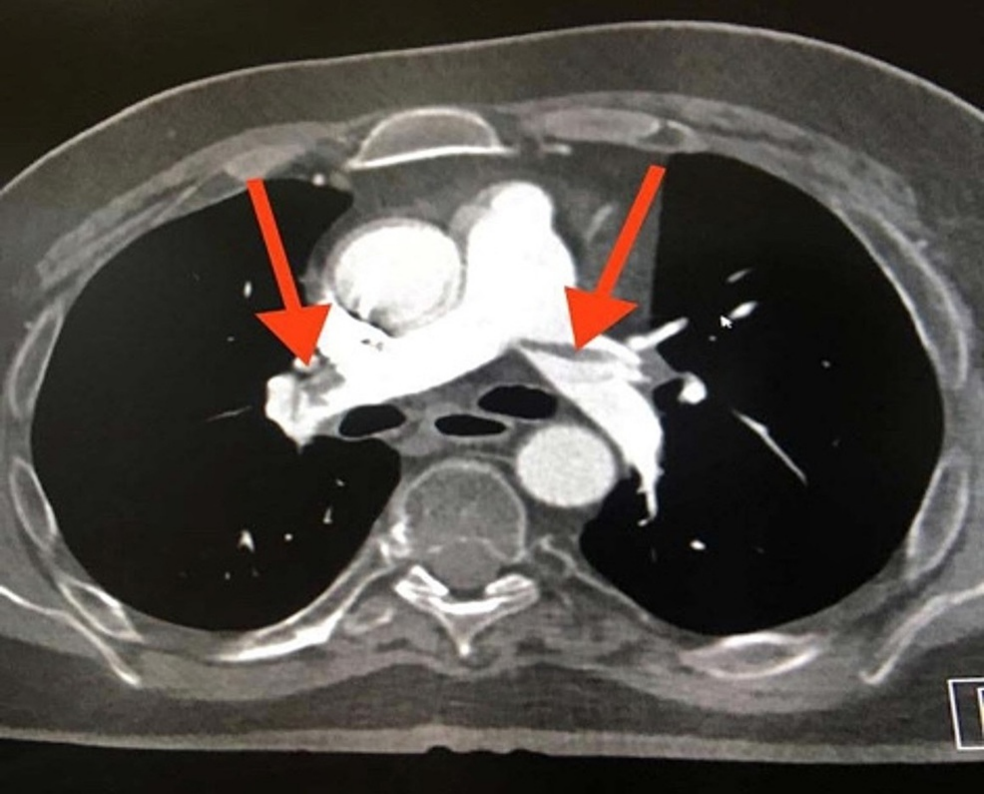
A cut of CT pulmonary angiography image showing filling defects in the pulmonary vasculature. A saddle embolus in the pulmonary artery extending into the left and right main pulmonary artery. Large embolus of interlobar, lobar, segmental, subsegmental left and right pulmonary arteries

A 31-year-old man was referred to our tertiary hospital with an initial impression of pulmonary embolism after initially presenting with shortness of breath and chest pain to another trauma center hospital. He had a previous history of fall and trauma that was investigated before and found to have features of a small fracture in the T9 vertebral body with no neurological effects. The patient had been having a prolonged period of immobility at home due to the trauma a few months before the presentation. CT pulmonary angiography was done that showed features consistent with saddle-shaped pulmonary embolism (Figure 1). The patient had been hemodynamically stable and had low-risk (class II) pulmonary embolism according to Pulmonary Embolism Severity Index scoring. He was referred for further management to our hospital.

Due to left lower limb swelling, a venous Doppler ultrasound was done, which highlighted extensive acute thrombus in the left external iliac vein to saphenofemoral vein, common femoral vein, popliteal vein, major leg veins to involve the saphenofemoral junction, and the greater saphenous vein. He was started on therapeutic subcutaneous heparin injection. The next day he developed hemorrhagic shock secondary to upper gastrointestinal bleed and his hemoglobin dropped from 11 to 7 g/dL. He was resuscitated with IV fluids, three units of packed red blood cells, and started on noradrenaline. An emergent bedside esophago-gastro-duodenoscopy was done that revealed a pyloric ulcer with a visible vessel, which was treated with the application of two hemoclips to achieve hemostasis.

**Figure 2:**
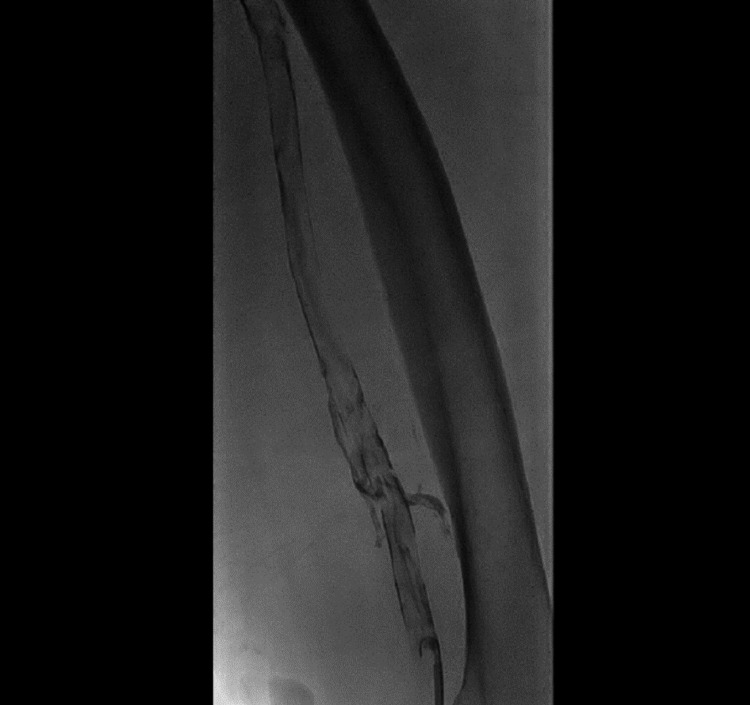
Left lower limb venogram showing extensive acute thrombosis involving left popliteal and femoral veins

**Figure 3:**
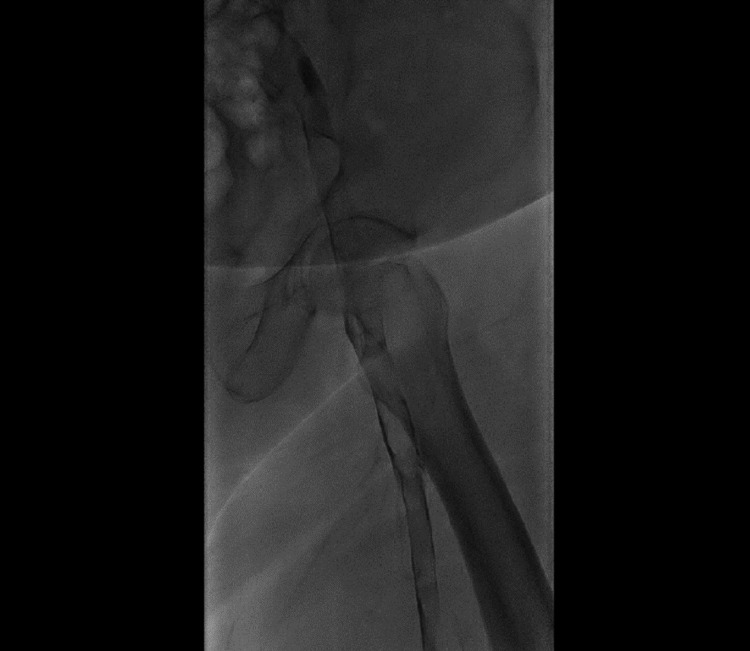
Left lower limb venogram showing extensive acute thrombosis involving left iliac vein

**Figure 4:**
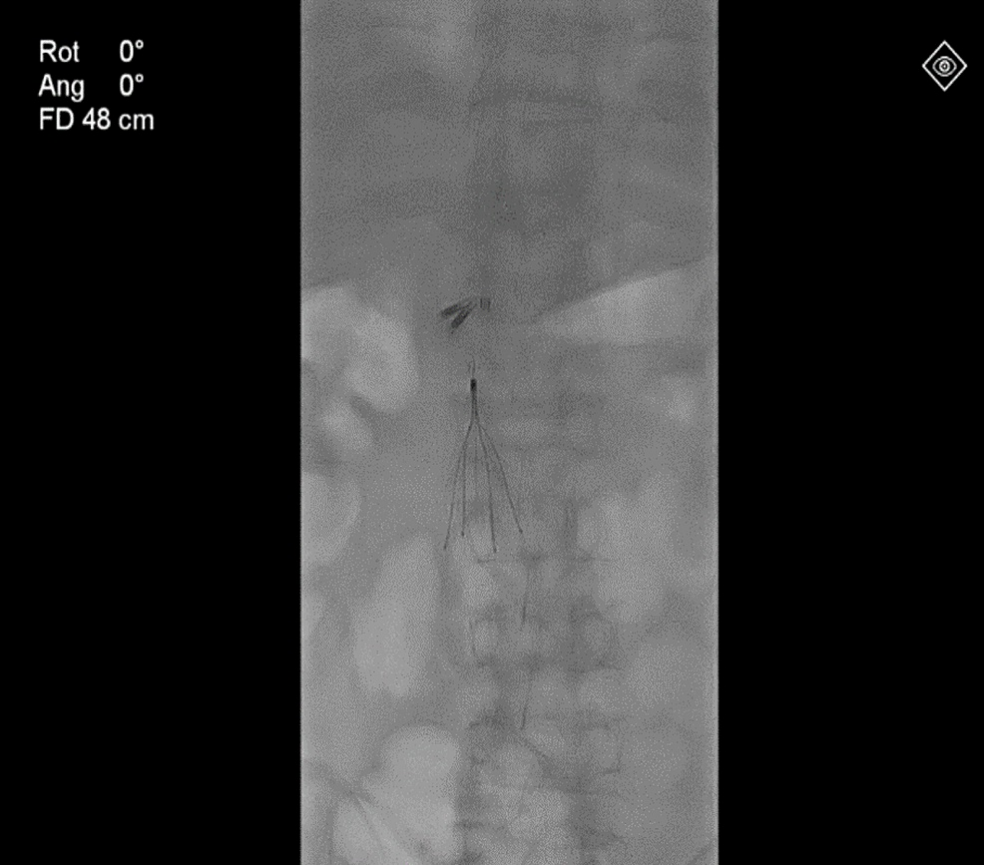
As a precaution to prevent clots migration to pulmonary circulation, an inferior vena cava filter was placed to prevent pulmonary embolism

Once the patient was stabilized, he was started on unfractionated heparin infusion and planned for contrast venography (Figures 2, 3) and inferior vena cava (IVC) filter placement (Figure 4). Clot aspiration was performed for the DVT followed by catheter-directed thrombolysis with alteplase 0.5 mg/h infusion overnight along with heparin infusion adjusted according to the activated partial thromboplastin time as per the vascular protocol. The patient was called in the following day for a repeat venogram that showed significant resolution of the femoral and iliac vein thrombosis.

**Figure 5:**
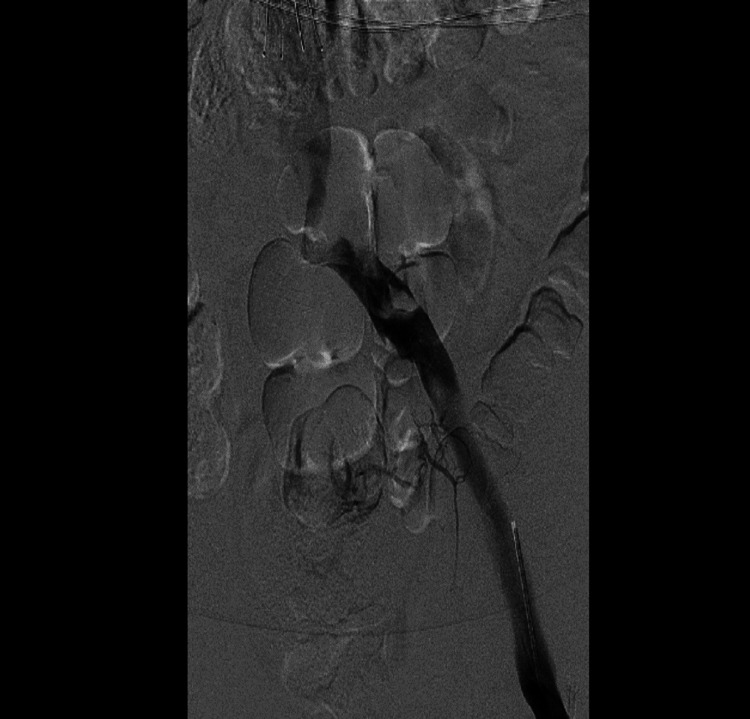
Venogram done 24 h after catheter-directed thrombolysis showing almost complete resolution of the deep vein thrombosis with good flow re-established in the femoral and iliac veins. There is, however, significant stenosis seen in the left common iliac vein suggestive of May-Thurner syndrome

**Figure 6:**
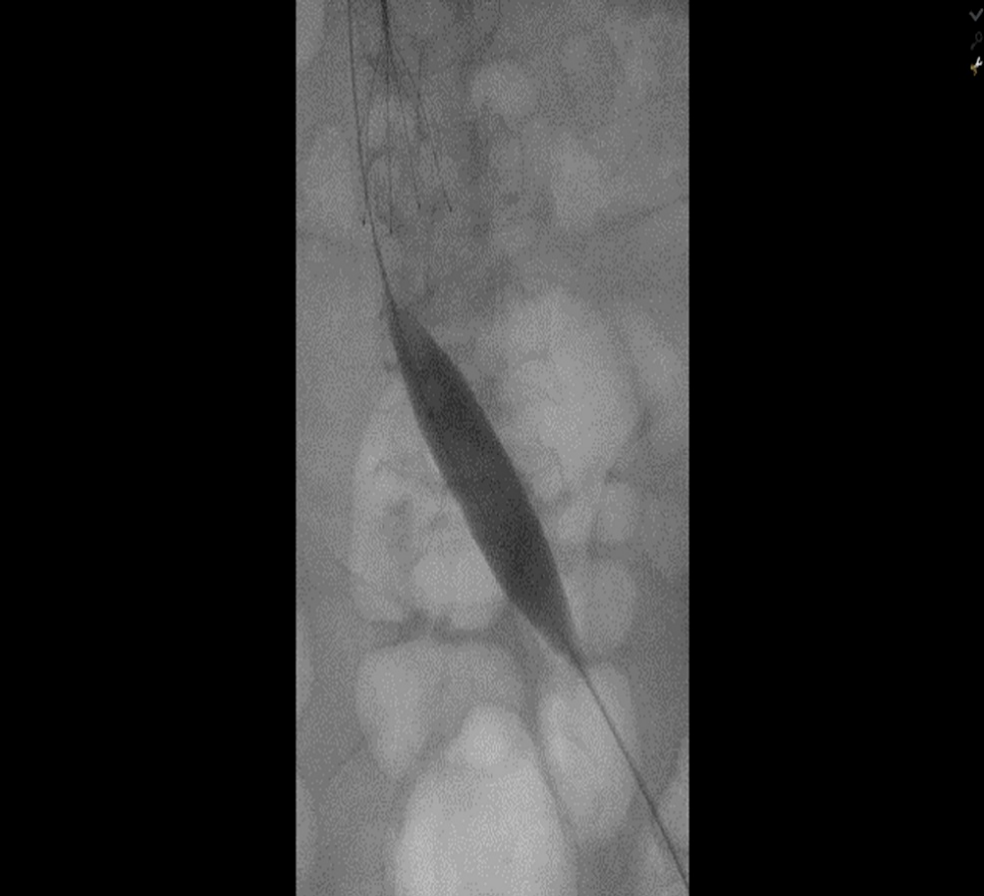
Balloon angioplasty

**Figure 7:**
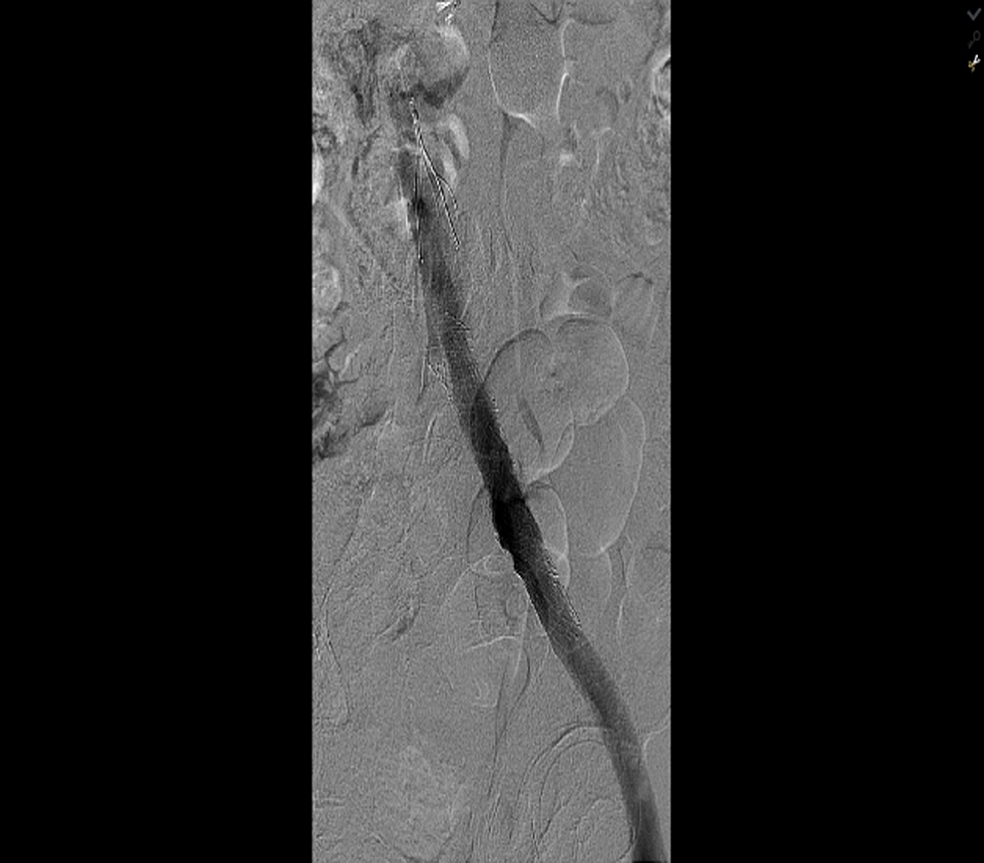
Iliac vein stenting with good results and no significant post-stenting residual stenosis

A good flow was established but there were still residual clots in the left common iliac, popliteal, and tibial veins. Therefore, it was decided to gain retrograde access toward the left popliteal and tibial veins to continue thrombolysis in a criss-cross fashion; that is, the dose of the alteplase was split into 0.2 mg/h through a catheter to the left common iliac vein and 0.3 mg/h through a catheter to the left popliteal and tibial veins. The patient was left for a couple of hours to be called back again for a repeat venogram. This time there was a significant resolution of the clot; however, there was still chronic thrombus and stenosis of the left common iliac vein (Figure 5). Hence, the diagnosis of MTS was made, and balloon angioplasty was performed with the application of a self-expandable metallic stent. Post-stenting angiography highlighted excellent flow in the left iliac veins (Figures 6, 7). The patient was transferred to the ward for observation and subsequently discharged in a hemodynamically stable state and was able to mobilize after a period of aggressive physiotherapy. He was discharged on anticoagulation therapy over six months with rivaroxaban, with a follow-up appointment for IVC filter retrieval.

## Discussion

Rudolf Virchow discovered an increased incidence of DVT in the left side of the lower limbs due to a compression by the right common iliac artery in 1851 [1]. Fifty years later, a study involving 107 cadavers showed a 32.7% presence of an obstruction in the left lower limb venous system [2]. Furthermore, a study done in 1943 on 399 cadavers demonstrated an almost similar prevalence of obstructions; however, it was discovered that obliteration was caused by deposition of collagen and elastin, which highlighted the possibility of an acquired defect rather than of being a congenital defect [3]. Perhaps the first detailed study was conducted by May and Thurner in 1957, where they studied 430 cadavers that were known to have left side lower limb DVT. They found a never-described-before anatomical defect of an overriding right common iliac artery obliterating the left common iliac vein by pressing it against the vertebral body in 22% of the cadavers, which they found also associated with “spur-like” projections inside the lumen of the veins at the site of the compression [1].

In the middle of the 19th century, Cocket and Thomas conducted the first study in living subjects presented with acute left-sided lower limb DVT and they investigated in detail the causes using venography, where they first described the nature of the obstruction as induration and ulceration that did not respond to a surgical mobilization of the nearby blood vessels, which suggested further intraluminal pathology rather than solely mechanical obstruction due to compression. They proposed that the presence of asymptomatic carriers of the defect as the chronicity of the anatomical anomaly eventually can lead to the formation of collaterals [1]. Over the years multiple studies demonstrated a possible prevalence of MTS between 4% and 32% in the normal population [4].

MTS, also known as Cocket’s syndrome or iliac vein compression syndrome, is a compression of the left common iliac vein by an overlying right common iliac artery against the vertebral body, most commonly the fifth lumbar vertebral body. The persistent pulsation from the artery causes tedious endothelial damage of the venous system with stasis of blood at the site of the compression. Subsequently, the process of damage and repair cycle can lead eventually to the deposition of collagen and elastin leading to intraluminal spur-like projection leading to a more permanent obstruction of the venous blood flow [1].

MTS was thought to be causing 2-3% of all DVTs; however, it was thought that MTS was underdiagnosed and under-recognized compared to other causes of venous thromboembolism like oral contraceptives, inherited thrombophilia, immobilizations, pregnancy, and recent surgery itself, as these could also represent the trigger factors of asymptomatic MTS to have thrombosis [5]. Unlike other causes of DVT, studies showed that MTS is more common in young females, possibly due to the different pelvis anatomy. It has been established that MTS is more among young individuals and DVT is usually triggered in those patients by prolonged immobility like in our case. Other triggers include pregnancy, postoperative period, and postpartum period [1].

The majority of patients with MTS are asymptomatic or have nonspecific symptoms; thus, the defect may go unrecognized until further serious complications occur. It is estimated that about 70% of patients are either asymptomatic or have vague symptoms. To emphasize, symptoms may include chronic unilateral swelling that is relieved by bed rest, venous claudication, varicose veins or even varicosities, venous ulcers, and superficial thrombophlebitis [6,7]. Therefore, MTS is more likely to present late with lower limb DVT, pulmonary embolism, and in rarer occasions as stroke in patients with patent foramen ovale [8]. Another rare presentation of the syndrome is phlegmasia cerulea dolens. The three stages of the disease start with an asymptomatic compression followed by the formation of endothelial hyperplasia accompanied by mild symptoms. The final stage is provoked by an earlier-mentioned trigger causing thrombosis [7].

MTS was diagnosed radiologically with gold standard venography. However, other modalities have been also used with good sensitivities, specificities, and less invasiveness including plain CT, CT venography, magnetic resonance venography (MRV), and to a more invasive extent intravenous ultrasonography (IVUS) [1,8,9,10]. US Doppler alone has low sensitivity in detecting such lesions located in the upper segment of the lower limb venous system [10]. Additionally, the high velocity of blood flow in the larger venous system carries a higher rate of false-positive findings of thrombosis and obstruction [1]. Thus, this makes US Doppler solely a poor detection method that can lead to a lower rate of detection and lead to an increased rate of complications subsequently [11].

CT venography, on the other hand, has been proven to be a useful tool with excellent sensitivity and specificity of 95%. It also offers the additional benefit of detecting other causes of external compression, such as localized hematoma, tumors, or lymph nodes. In contrast, the usefulness drops in patients with dehydration as it can give false-positive results [12]. The compression morphology in the CT venography is classified into three types including type I, which represents a point of compression of the left common iliac vein at the site of crossing under the origin of the right common lilac artery. Type II, where there is atrophy of the segment of the left common iliac vein from the site of the compression to the division site where the vein divides into internal and external iliac veins. Type III is a cord-like obliteration of the left common iliac vein [13]. In contrast, MRV offers no additional benefits over CT venography and is of limited use due to the cost [14].

The gold standard for MTS diagnosis is using venography with or without the IVUS. The typical findings in venography are collaterals and pressure gradient of more than 2 mmHg across the stenosis at rest [1]. Moreover, venography can provide useful information about the morphology and the percentage of the diameter occluded by the compression, as well as details about the chronicity of the lesion [15]. However, there is no specific pre-determined percentage of stenosis after which the lesion is thought to be symptomatic. Multiple studies have shown variable results with lesions above 50-70% stenosis found to have more symptoms compared to a lower range of occlusions; however, DVT happened in a lower percentage of stenosis [1]. A study done in 2017 showed that for each 10% increase in stenosis the odds of DVT risk increased by 2.18. The addition of IVUS can increase the accuracy of stent positioning and reduce the risk of contrast-induced nephropathy or allergies caused by the contrasts used in traditional venography [16].

The mainstay treatment of thrombosis in settings of MTS consists of three major levels of management. First is preventing the progression of the DVT by full anticoagulation, followed by catheter-directed thrombolysis or thrombectomy and finally correction of the physical defect by stenting or other surgical options including bypass. Over the years the medical treatment alone has proven to be less effective without additional endovascular intervention and was associated with suboptimal outcomes, perhaps due to the nature of the root cause of the disease, the pulsatile mechanical obstruction [2]. Burger and colleagues reported the first efficacy evaluation of catheter-directed thrombolysis and endovascular stenting in MTS. Insertion of IVC filter was associated with more favorable outcomes, especially in preventing distal embolization such as pulmonary embolism [1]. Thus, guidelines from the International Radiology Society and Society of Vascular Surgery recommend direct catheter thrombolysis using tissue plasminogen activator or urokinase infused through the tip of the catheter for a duration of 24-48 h followed by thrombectomy with angioplasty if indicated and then stenting [17]. Left iliac vein stenting has been associated with almost no mortality, trivial morbidity, a low rate of in-stent restenosis, and excellent long-term patency reaching to 95-100% two-year patency rate [1,2]. As the surgical option is more invasive and associated with higher morbidity with additional anesthesia risk, it is less favorable and is indicated only when the radiological intervention fails [18]. Surgical options include surgical thrombectomy combined with anatomical correction. Common procedures include saphenofemoral cross-over bypass, cross pelvic venous bypass, femoro-femoral bypass, femoro-caval bypass, ilio-iliac bypass, and aortic elevation procedure [2]. Studies have confirmed that thrombectomy alone was associated with a 70% rate of re-occlusion during follow-up [10].

Recent recommendation advocates for three to six months of systemic anticoagulation after stenting for MTS [2]. Data from the trials ATTRACT and CaVent, which compared catheter-directed thrombolysis with stenting followed by systemic anticoagulation, demonstrated better outcomes in comparison with anticoagulation alone. Low-molecular-weight heparin or fondaparinux is usually preferred as initial anticoagulation over unfractionated heparin due to the risk of heparin-induced thrombocytopenia [19]. Guidelines recommend vitamin K antagonists as long-term anticoagulants such as warfarin after stenting. Inhibitors of factor Xa also have been approved for DVT treatment, and agents may include rivaroxaban and apixaban [20]. A randomized control trial that evaluated the safety of rivaroxaban compared with warfarin in patients with iliofemoral DVT where 50% of them had MTS showed that rivaroxaban was associated with a lower risk of gastrointestinal bleeding. In our case, the patient was discharged home on rivaroxaban with follow-up appointments. These results also were confirmed by a study done by Sabastian and colleagues, which showed similar results [19].

The delay in identifying MTS increases the risk of recurrent DVTs and promotes progression to more life-threatening complications, such as pulmonary embolism. Studies have found that delay in anticoagulation increased the risk of MTS [20]. Other complications of the syndrome may include iliac vein rupture, retroperitoneal hemorrhage, and chronic thrombosis, which is more challenging to treat [10]. Suboptimal thrombus treatment leading to residual thrombosis after thrombolysis increases the risk of post-thrombotic syndrome [1]. Other complications could be associated with intervention, including stent migration, thrombosis, stent fracture, and hemorrhage or other medication side-effects [8].

## Conclusions

MTS is a rare cause of venous thromboembolism caused by compression of the left common iliac vein by the right common iliac artery against the nearby vertebral bodies. We reported this case of complicated course that started with features of pulmonary embolism initially. The early recognition of MTS aids in proper management that can prevent fatal complications. Once recognized, MTS is treated with catheter-directed thrombolysis followed by correction of the anatomical defect by stenting. Anticoagulation after stenting was recommended and was associated with a better outcome. This case report enlightens the need for a proper evaluation to recognize rare causes of common presentations.
